# Protein Corona Attenuates the Targeting of Antitumor Sialyl Lewis X-Decorated Liposomes to Vascular Endothelial Cells under Flow Conditions

**DOI:** 10.3390/pharmaceutics15061754

**Published:** 2023-06-16

**Authors:** Natalia R. Onishchenko, Alexey A. Moskovtsev, Maria K. Kobanenko, Daria S. Tretiakova, Anna S. Alekseeva, Dmitry V. Kolesov, Anna A. Mikryukova, Ivan A. Boldyrev, Marina R. Kapkaeva, Olga N. Shcheglovitova, Nicolai V. Bovin, Aslan A. Kubatiev, Olga V. Tikhonova, Elena L. Vodovozova

**Affiliations:** 1 Shemyakin–Ovchinnikov Institute of Bioorganic Chemistry, Russian Academy of Sciences, ul. Miklukho-Maklaya 16/10, 117997 Moscow, Russia; natalia.r.onishchenko@gmail.com (N.R.O.);; 2 Institute of General Pathology and Pathophysiology, Russian Academy of Sciences, ul. Baltiyskaya 8, 125315 Moscow, Russia; 3 N.F. Gamaleya National Research Center for Epidemiology and Microbiology, Ministry of Healthcare of the Russian Federation, ul. Gamaleya 18, 123098 Moscow, Russia; 4 Institute of Biomedical Chemistry, ul. Pogodinskaya 10, 119121 Moscow, Russia

**Keywords:** nanosized liposomes, lipophilic prodrug, melphalan, Sialyl Lewis X, endothelial cells, microfluidics, proteome

## Abstract

Previously, we showed in the human umbilical vein endothelial cells (HUVECs) model that a liposome formulation of melphalan lipophilic prodrug (MlphDG) decorated with selectin ligand tetrasaccharide Sialyl Lewis X (SiaLe^X^) undergoes specific uptake by activated cells and in an in vivo tumor model causes a severe antivascular effect. Here, we cultured HUVECs in a microfluidic chip and then applied the liposome formulations to study their interactions with the cells in situ under hydrodynamic conditions close to capillary blood flow using confocal fluorescent microscopy. The incorporation of 5 to 10% SiaLe^X^ conjugate in the bilayer of MlphDG liposomes increased their consumption exclusively by activated endotheliocytes. The increase of serum concentration from 20 to 100% in the flow resulted in lower liposome uptake by the cells. To elucidate the possible roles of plasma proteins in the liposome–cell interactions, liposome protein coronas were isolated and analyzed by shotgun proteomics and immunoblotting of selected proteins. Proteomic analysis showed that a gradual increase in SiaLe^X^ content correlated with the overall enrichment of the liposome-associated proteins with several apolipoproteins, including the most positively charged one, ApoC1, and serum amyloid A4, associated with inflammation, on the one hand, and a decrease in the content of bound immunoglobulins, on the other. The article discusses the potential interference of the proteins in the binding of liposomes to selectins of endothelial cells.

## 1. Introduction

Today, liposomal formulations of anticancer drugs are used to treat a whole number of tumor diseases in clinics [1,2,3,4]. Parenteral administration of drug-loaded nanosized liposomes permits the alleviation of systemic toxicity of small-molecule antitumorials through lower concentration of the free drug in blood and tendency of nanoparticles to accumulate in tumors’ impaired vasculature (enhanced permeability and retention effect, EPR) [5,6]. Yet ligand-mediated targeting of tumors continues to be a challenge owing, first of all, to the vascular and interstitial barriers [1,7,8]. Further, spatial and temporal heterogeneity in tumor tissue caused by the growth of multiple genetic subpopulations prevents the success of therapy that specifically targets individual clones of malignant cells [9]. Targeting the cells of angiogenic endothelium, which support the survival and growth of tumor tissue, could resolve these obstacles. The strategies include targeting vascular endothelial growth factor/receptors [10,11,12], αvβ3 integrin receptors overexpressed on endothelial cells in various types of cancer [13,14,15], as well as vascular cell adhesion molecule-1 (VCAM-1) [16,17,18,19] and intercellular adhesion molecule-1 (ICAM-1) [20,21].

Selectins (carbohydrate-binding cell adhesion molecules) have evolved as a promising target for delivery to the tumor endothelium [22,23]. They are expressed on the surface of activated endothelial cells (E- and P-selectins), circulating leukocytes (L-selectin), and activated platelets (P-selectin). Selectins play a key role in inflammatory reactions and the development of metastases, according to a mechanism that sequentially includes the events of leukocyte rolling, their firm adhesion, and trans-migration through the endothelium. [24,25]. Selectin ligands include a variety of sialylated and fucosylated glycans containing tetrasaccharide Sialyl Lewis X (SiaLe^X^, Neu5Acα2-3Galβ1-4(Fucα1-3)GlcNAcβ) as a common epitope [26]. Several studies have been reported on drug delivery to tumors and inflammatory foci using immunoliposomes with engrafted anti-E-selectin mAbs (for example, [27]; reviewed in [22]). SiaLe^X^ conjugated onto liposome surface has also been used for targeting activated endothelial cells [28]. The complexity of synthesis stimulated studies with liposomes decorated with a simpler SiaLe^X^ glycomimetic [29,30].

**Figure 1:**
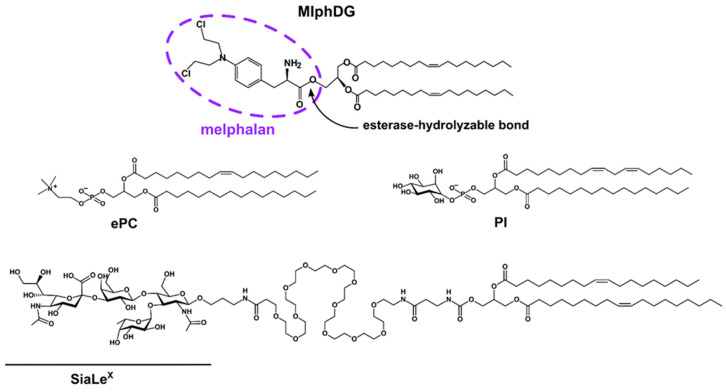
Structures of melphalan lipophilic prodrug (MlphDG), SiaLe^X^-conjugate, and representative structures of soybean phosphatidylinositol (PI) and egg phosphatidylcholine (ePC) used for liposome preparation.

Previously, we showed that liposomal formulation of a melphalan lipophilic prodrug (MlphDG, Figure 1) could cause severe injuries to tumor vessels in the Lewis lung carcinoma model when equipped with a diglyceride conjugate of SiaLe^X^ in the bilayer [31]. It is most likely that endothelial cells were disrupted by the cytotoxic action of melphalan generated intracellularly, since prodrug-free SiaLe^X^-liposomes did not cause vascular damage [31]. Our further study on the human umbilical vein endothelial cell (HUVEC) model revealed specific interactions between SiaLe^X^-targeted MlphDG liposomes and activated HUVECs expressing E-selectin on their surface, which resulted in rapid internalization of the liposomes [32]. Concurrent destabilization of the liposome membranes presumably facilitates esterase hydrolysis of the lipophilic prodrug with the release of the cytotoxic agent. Importantly, resting HUVECs (i.e., not stimulated by a cytokine) bound only a small number of SiaLe^X^-liposomes, which kept the membrane intact [32]. Thus, the approach promises selective delivery of drug-loaded liposomes to the angiogenic endothelium of tumors.

Our liposomal formulation bears a significant amount of phosphatidylinositol (PI) equal to the MlphDG load. PI can increase the circulation half-life of nanosized liposomes, presumably due to its voluminous, highly hydrated head group [33,34]. Pegylation of liposomes, widely used to stabilize them in the bloodstream, often causes adverse reactions against PEG (reviewed in [35,36]). Recently, we showed that an equimolar amount of PI and MlphDG favors liposome stability and integrity under the impact of plasma proteins [37,38]. The formulation showed good hemocompatibility and did not activate the complement system, either without SiaLe^X^-conjugate in the bilayer or with it [39].

In complex in vivo environments, a number of factors interfere with targeting. Particularly, hydrodynamic parameters are key elements of the endotheliocyte environment, yet their effects on the uptake of delivery vehicles are poorly studied. Another important factor affecting the interactions of nanoparticles with cells is the protein corona, that is, a layer of proteins and lipoproteins that instantly covers the carrier in the presence of serum (or plasma) proteins, under both static and flow conditions [40,41,42]. Pioneering works on the effect of flow conditions on liposome protein corona reported differences in the compositions of proteins deposited on pegylated cationic liposomes [43], as well as the internalization rate thereof by cancerous cells [44] under static and flow conditions. Several studies utilized microfluidic chips for precise control of conditions under which the nanoparticles and biologic fluids interact under flow [45,46]. Very few studies proposed the microfluidics approach for simultaneous in-flow corona generation on nanoparticles and their interaction with cells [47,48].

The goal of this work was to study the binding capacity of the SiaLe^X^ liposomal formulation of MlphDG to endothelial cells in a model of blood flow. We used a microfluidic device that allowed us to create controlled hydrodynamic conditions for cultured endothelial cells and demonstrated the persistence of increased absorption of SiaLe^X^ liposomes by activated HUVECs with an increase in SiaLe^X^ ligand content. Liposome–cell interactions were probed in human serum flow. To take into account the possible contributions of the protein corona to liposome–cell interactions, we isolated liposome–protein complexes formed during the incubation of MlphDG liposomes in plasma and studied corona proteins by methods of classical biochemistry and modern proteomics. Proteomic analysis revealed differences between targeted and plain samples, which could be the reason for the weakening of liposome–cell interactions observed in the presence of plasma proteins.

## 2. Materials and Methods

### 2.1. Chemicals and Materials

Phosphatidylcholine from egg yolk (ePC; USP grade, Lipoid E PC S) was purchased from Lipoid GmbH (Heidelberg, Germany); raw soybean phosphatidylinositol (PI, a kind gift from Lipoid) was purified by chromatography on silica gel and characterized by ^1^H-NMR spectroscopy as an individual phospholipid. Dioleoylglycerol ester melphalan conjugate (MlphDG) [49], diglyceride conjugate of SiaLe^X^ [50] and 1,3,5,7-tetramethyl-BODIPY-labeled phosphatidylcholine (TMB-PC) [51] were synthesized as previously reported. Bovine serum albumin (BSA) and Tris were purchased from PanEco (Russia), Sepharose CL-4B for size-exclusion chromatography (SEC), from Sigma (Burlington, MA, USA). The solvents were purified by distillation.

Buffer compositions were as follows: phosphate buffered saline (PBS; KH_2_PO_4_, 0.2 g/L; NaH_2_PO_4_ × 2H_2_O, 0.15 g/L; Na_2_HPO_4_, 1.0 g/L; KCl, 0.2 g/L; NaCl, 8.0 g/L, pH 7.4); Tris-buffered saline (TBS; NaCl, 4.39 g; Tris, 3.03 g; H_2_Odd, 500 mL), pH 7.97; Tris-HCl, pH 7.0 (30 mM Tris); SDS-PAGE sample buffer (0.075 M Tris-HCl, pH 6.8, 10% glycerin, 2% SDS, 5% β-mercaptoethanol, 0.01% bromophenol blue).

Primary polyclonal rabbit antibodies to human component C3 (Cloud-Clone Corp., Houston, TX, USA), rabbit antibodies to human vitronectin and complement factor I (Complement Tech., Tyler, TX, USA), sheep polyclonal antibodies to human C4BP (Abcam, Cambridge, UK), monoclonal murine antibodies to immunoglobulins G and M, ApoA1 (IMTEK, Moscow, Russia), and polyclonal rabbit antibodies to human ApoH (Cloud-Clone Corp., Houston, TX, USA) were used. Rabbit antibodies to sheep IgG conjugated with horse-radish peroxidase (Jackson ImmunoResearch, West Grove, PA, USA) and rabbit antibodies to mouse IgG and mouse antibodies to rabbit IgG conjugated with horse-radish peroxidase (Sigma-Aldrich, St. Louis, MO, USA) were used as secondary antibodies.

Blood samples from nine healthy donor volunteers were collected in vacuum tubes over EDTA (Greiner Bio-One, Kremsmünster, Austria). The plasma was separated by centrifugation at room temperature for 30 min at 1660× *g* (CM-6M, ELMI, Riga, Latvia). The supernatants were pooled, transferred into fresh tubes, and centrifuged at 600× *g* for another 10 min. Plasma aliquots were frozen in liquid nitrogen and stored at −70 °C.

In order to obtain the serum for microfluidic experiments, Becton Dickinson vacutainers with a coagulation activator were used. The serum was frozen in liquid nitrogen and thawed at 37 °C immediately before usage. Human recombinant tumor necrosis factor alpha (TNF-α) was a kind gift received from Dr. L.N. Shingarova (IBCh RAS).

### 2.2. Preparation of Liposomes

Liposomes (large unilamellar vesicles) were prepared by lipid film hydration followed by extrusion as described earlier [37,52]. Mixtures of ePC, PI, SiaLe^X^-conjugate and MlphDG in the required molar ratios (Table 1) in chloroform–methanol (2:1) were dried by rotary evaporation in round-bottomed tubes and then in vacuum at 7 Pa for at least 1.5 h. The lipid films were hydrated with PBS (unless otherwise specified) under shaking at room temperature for 2 h. Then, the suspensions were subjected to 5–7 cycles of freezing/thawing (N_2_ liquid/+40 °C) and extruded 20 times through polycarbonate Whatman Nuclepore membrane filters (Cytiva, Marlborough, MA, USA) with a pore size of 100 nm using a Mini-Extruder setup (Avanti Polar Lipids, Alabaster, AL, USA). The resulting dispersions were stored at 4 °C and used for experiments within 3 days.

Phospholipid concentrations in liposome dispersions were measured by the enzymatic colorimetric phosphatidylcholine assay (Sentinel Diagnostics, Milan, Italy), as described in [52]. Prodrug concentrations were measured by UV spectrophotometry after liposome disruption with ethanol (λ_max_ MlphDG 260 nm, ε 16,100 M^−1^cm^−1^).

To obtain fluorescently labeled liposomes for microfluidic experiments, 0.5 mol % TMB-PC was added at the stage of lipid film formation.

### 2.3. Liposome Hydrodynamic Diameter and Zeta Potential Measurements

Hydrodynamic diameters of the liposomes were measured in diluted dispersions (final lipid concentration 50 µg/mL in PBS) using a Brookhaven Particle Analyzer 90+ (Brookhaven Instruments Corp., Holtsville, NY, USA; helium-neon laser, 633 nm, 90° angle), 3 cycles of 1 min.

For reliable measurements of zeta potential according to the criteria of the Smoluchovski model [53], liposome samples with diameters of around 200 nm were prepared in 10 mM KCl, 1 mM K_2_HPO_4_, 1 mM KH_2_PO_4_, pH 7.0 buffer (PB) using 200 nm polycarbonate membrane filters for extrusion. Samples of the liposomes (0.85 mL, 1 mg/mL total lipids) were equilibrated for 1 min in cuvettes before measurements of 100 to 500 cycles per sample were performed at 25 °C using Litesizer 500 (Anton Paar GmbH, Austria; 658 nm). Measurements were completed in triplicate.

### 2.4. Determination of the Zeta Potential of Liposome–Protein Complexes

Liposomes of approximately 200 nm in size were prepared as described for the measurements of zeta potential in Section 2.3. A 200-μL aliquot of frozen pooled plasma was thawed on a water bath at 37 °C for 15 min and centrifuged at 12,000× *g* for 30 min. Then, 90 μL of supernatant was mixed with 90 μL liposome dispersion (20 mM) and incubated at 37 °C for 15 min. Proteolysis was stopped by adding ethanol solution of phenylmethylsulfonyl fluoride (0.1 M, 1.8 μL) and 180 μL of thus obtained dispersion was applied onto a Sepharose CL-4B column (1.0 × 27 cm) equilibrated with PB. After elution of the void volume (~7 mL), 16 fractions of ~200 µL were collected. Liposome and protein elution was monitored by absorbance at 210 and 280 nm using the NanoDrop One^C^ spectrophotometer (Thermo Fisher Scientific, Waltham, MA, USA). Five fractions, most rich with liposomes, were pooled, mixed, and transferred to a disposable Omega cuvette (Anton Paar GmbH, Austria) without dilution to obtain a zeta-potential value. For each liposome sample, incubation and further treatment were performed thrice.

### 2.5. Hydrodynamic Modeling: Fabrication of Microfluidic Chips

**Figure 2:**
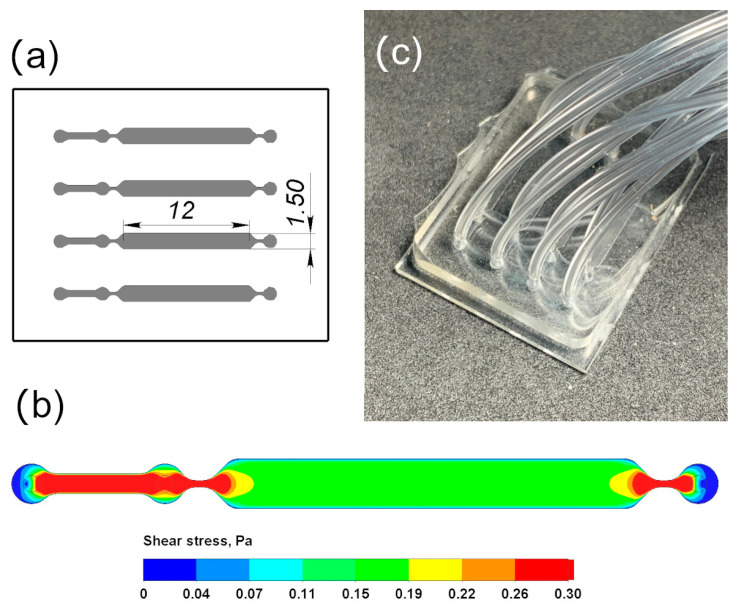
(**a**) The scheme of the microfluidic chip: channel width 1.5 mm, height 0.2 mm, length 12 mm. (**b**) Heat map of the shear stress distribution in the chip channel at a volumetric velocity of 100 µL/min. (**c**) Photo of the microfluidic chip.

Microfluidic chips were produced using standard soft lithographic techniques [54]. Chip configuration was first designed in CAD. The chip consisted of four microchannels with a broad middle part (see Figure 2a for the scheme). The broad part was 1.5 mm wide and approximately 12 mm long. The height of all channels was 200 µm. Each channel had three inlets: one for cell loading and bubble elimination and two for medium flow. Soft lithography molds were micro-milled from plexiglass. We used polydimetisiloxane (PDMS, Sylgard 184 Silicone Elastomer Kit, Dow Corning, Midland, MI, USA) as a material for chip fabrication, with cover glass as a substrate. The prepolymer mixture was prepared and cured according to the manufacturer’s instructions. Cured replicas were bonded to glass substrates upon activation with oxygen plasma (Atto, Diener Electronic, Ebhausen, Germany). Computational fluid dynamics analysis was used for the estimation of applied shear stress. Volume flow rate and total atmospheric pressure were used as boundary conditions for the inlet and outlet, respectively.

### 2.6. Primary Culture of Human Umbilical Vein Endothelial Cells

Individual donor endothelial cells (HUVECs) were isolates from human umbilical cords according to the methods of Jaffe [55] and Scheglovitova [56]. Umbilical cords were obtained after normal parturition from five healthy donors following informed written consent.

Briefly, fresh umbilical veins were cannulated and filled with dispase solution (2 mg/mL) (Gibco, New York, NY, USA) and incubated at 37 °C for 30 min. Then, the veins were perfused with PBS. Cells were collected from the perfusate by centrifugation at 1000 rpm for 10 min, resuspended in Medium 199 (Gibco) supplemented with 10% fetal calf serum (HighClone, Logan, UT, USA), 200 μg/mL endothelial growth factor (Sigma), 100 μg/mL heparin (Moskovskii endokrinnyi zavod, Moscow, Russia), and 50 μg/mL gentamycin (KRKA), and seeded into 25–75 sm^2^ (6-well plates) cultural flasks. Cells were cultured in a humidified atmosphere of 5% CO_2_ at 37 °C. The confluent primary monolayers were washed and trypsinized (0.05% trypsin + 0.02% EDTA, Gibco). The cells were resuspended into a complete medium, seeded in 48-well plates (140,000 cells/mL) (Costar, Washington, DC, USA), and cultured for four days. Only the first subcultures were used for further experiments.

### 2.7. Cell Culturing in Microfluidic Chips

In order to form a monolayer of HUVECs in a microfluidic chip, the confluent primary monolayers were washed and trypsinized (0.05% trypsin + 0.02% EDTA, Gibco). The cells were resuspended into a complete medium, and a suspension of 5 × 10^6^ cells/mL was injected into the channels of the chip precoated with 0.01% solution of collagen overnight at room temperature, after which the chip was placed in a wet chamber in the incubator overnight.

The monolayers of HUVECs on the walls of microchannels were inspected under an inverted microscope. The chip was connected to a dosing system, Nemesis (Cetoni, Korbußen, Germany), which allows precise computer control of the flow rate with Teflon tubes. Temperature control in the chip channels was carried out using a thermoelectric module guided by a controller (RMT Ltd., Moscow, Russia) with a temperature feedback system.

### 2.8. Liposome Uptake by HUVECs in Microfluidic Chips under Flow

Cells in the chip were activated with 50 ng/mL TNF-α for 4 h at 37 °C before the flow was applied.

Human blood serum or DMEM medium without phenol red (Fluorobrite) with 20% FBS was mixed with fluorescently labeled liposomes to a final concentration of 50 µM by lipids and then loaded into the syringes of the Nemesis dosing system. The mixture was fed to fill the tubes leading to the chip. After that, the tubes were connected to the ports of the chip and the flow was started into microchannels with a given volume flow rate. We used non-pulsating constant unidirectional laminar flow with a flow rate of 100 μL/min in the channels of the microchip. As a control, we used a flow of 0 μL/min, also referred to as “no flow”. The no flow conditions mean that after the introduction of the liposome suspension into the microchannel, there was no flow in this microchannel during the entire time of the experimental exposure. We also used quasi-static conditions with the flow of 5 µL/min to simulate a very low shear stress (<0.01 Pa), resembling the interstitial flow-induced physiological shear of cells [57]. After 15 min of exposure to the flow, a 4% solution of formalin in PBS was injected into the microchannels to fix the cells for 5 min. Then, the channels were washed with 2 mL of PBS per channel.

For the subsequent segmentation of the image of the cell monolayer, labeling with propidium iodide (PI, 1 µg/mL) and a lipophilic cationic dye 1,1’-dioctadecyl-3,3,3’,3’-tetramethylindocarbocyanine perchlorate (DiI, 1 μM) was used. These probes were injected sequentially into microchannels with pre-fixed monolayers of HUVEC cells.

The microchannels of the chip were visualized on a Nikon Eclipse TE 2000-u C1 confocal inverted microscope using the EZ-C1 software version 3.91. The images were captured sequentially using 488 and 543 nm lasers and 515/30 nm and 570 LP detectors, respectively, with 4×, 10×, and 20× Plan Fluor objectives. The parameters of image acquisition (gain, exposure time, and laser power) were not changed within a series of experiments but could vary between the series. Furthermore, 16-Bit monochrome images were segmented using an ImageJ script and the mean fluorescence intensity of individual cells was calculated. At least 3 fields were recorded with approximately 2700 cells per field.

In order to select the method of statistical analysis, the data were first checked for normal distribution using the Kolmogorov–Smirnov test. The distributions of mean cell fluorescence intensities after incubation with fluorescently labeled liposomes in microfluidic chips under different experimental conditions were usually not normal, and nonparametric methods were used for their further analysis: the median test and the Kruskal–Wallis test. Statistical analysis was performed with the Statistica software version 10.0 (StatSoft Inc., Tulsa, OK, USA). Differences were considered significant at *p* < 0.05. The number of repeated experiments in each series was at least three.

### 2.9. Liposome Uptake by HUVECs under Static Conditions

For the experiments under static conditions, 10 mM total lipid liposomes with 1 mol % TMB-PC were used. A 600-μL aliquot of frozen pooled plasma was thawed on a water bath at 37 °C and centrifuged at 12,000× *g* for 30 min. Then, 540 μL of supernatant was mixed with 60 μL liposome dispersion and incubated at 37 °C for 15 min. Similarly, liposomes were incubated in 90% FBS when indicated. Immediately after that, the liposomes were dissolved in serum-free Medium 199 (PanEko, Russia) to 100 μM total lipids (final plasma concentration in the medium was ~10% by volume).

HUVECs confluent monolayer in 48-well plates (seeding density 140,000 cells/mL) was activated with 50 ng/mL TNF-α for 4 h at 37 °C, washed with DPBS (PBS with calcium and magnesium salts, PanEco, Russia), and incubated with liposomes (with protein corona) for various incubation periods. Then, the cells were washed with DPBS, resuspended in 0.02% EDTA solution (10 min, 37 °C) and then in 0.3 μg/mL PBS solution of propidium iodide, and stored on ice prior to measurements on the flow cytometer.

Cell suspensions were analyzed on a FACScan (Becton Dickinson, Franklin Lakes, NJ, USA) flow cytometer using the CellQuest software. The fluorescence signal was detected in channels FL1 (515–545 nm), FL2 (565–610 nm), and FL3 (>650 nm), two runs of 10,000 target events per sample. To exclude cell aggregates, debris, or dead cells from the analysis, target events were gated by forward and side scattering (FSC/SSC) and the propidium iodide signal.

### 2.10. Isolation of Liposome–Protein Complexes from Plasma

A 600-μL aliquot of frozen pooled plasma was thawed on a water bath at 37 °C for 15 min and centrifuged at 12,000× *g* for 30 min. Then, 540 μL of supernatant was mixed with 60 μL liposome dispersion (40 mM) and incubated at 37 °C for 15 min. After the incubation, proteolysis was stopped by adding an ethanol solution of phenylmethylsulfonyl fluoride (0.1 M, 6 μL) and 500 μL of thus obtained dispersion was applied onto a Sepharose CL-4B column (1.5 × 33 cm) equilibrated with PBS. After elution of most of the void volume (~15 mL), 16 fractions of ~1 mL were collected. Liposome and protein elution was monitored as described in Section 2.4. The four fractions that were most rich with liposomes were pooled and concentrated (in several aliquots) down to 120 μL using the Vivaspin 2 concentrators (MWCO 300 kDa, Sartorius, Göttingen, Germany) by centrifugation for ~100 min at 740× *g* (CM-6M, ELMI) at room temperature. Before usage, membranes of the concentrators were passivated with 1% BSA solution overnight at 4 °C and washed as specified by the manufacturer.

As a negative control (when no protein corona is formed), a plasma sample was treated in the same manner as the liposome–protein mixtures. Upon SEC separation of the plasma control, protein concentration in fractions corresponding to the liposome elution was too low for proteomics analysis (see further). For this reason, we combined the material of three SEC elutions of control plasma.

### 2.11. HPLC-MS/MS Sample Preparation

Samples of liposome–protein complexes were processed with trypsin according to the S-Trap™ (suspension trapping proteolysis) micro spin column [58]. All steps were performed according to the digestion protocol recommended by the manufacturer. Briefly, to the samples in the Eppendorf tubes (2 mL), 20 μL 100 mM triethylammonium bicarbonate (TEAB) buffer was added and mixed with lysing buffer (10% SDS, 100 mM TEAB, pH 7.55) with ratio 1:1. The dispersion was sonicated for 20 s thrice on ice and centrifuged at 13,000× *g* for 8 min at 10 °C for foam removing. Then, 2 μL 0.5 mM tris(2-carboxyethyl)phosphine hydrochloride (Sigma-Aldrich) and 4 μL 400 mM 2-chloroacetamide were added to the sample solution tubes. The samples were incubated for 30 min at 80 °C and cooled to room temperature. After the addition of 12% H_3_PO_4_ at a 1:10 ratio, the dispersion was pipetted, and 6-time volumes (304 μL) of S-Trap protein binding buffer (90% aqueous methanol containing 10 mM TEAB, pH 7.5) were added. The resulting mixture for each sample was shaken thoroughly and applied twice (by 152 μL) onto S-trap filters and centrifuged for 4 min at 4000× *g*. Then, each sample was washed with 150 μL S-Trap protein binding buffer 4 times at 4000× *g* with flow-through removal. Trypsin solution in 50 mM TEAB was added to the samples (trypsin to protein ratio of 1:25) and incubated for 1.5 h at 47 °C. After the incubation, 40 μL 50 TEAB and 0.2% formic acid were added to the S-trap filters and the samples were centrifuged for 4 min at 4000× *g*. Then, obtained peptides for each sample were eluted in the clean tubes with 35 μL 0.2% formic acid solution in 50% acetonitrile added and centrifuged for 4 min at 4000× *g*. The hydrolyzates were dried in a vacuum concentrator (Eppendorf Concentrator plus, Germany) and redissolved in 0.1% formic acid to a final concentration of 1 μg/μL. Peptide concentration in samples was assayed using the Pierce™ Quantitative Colorimetric Peptide Assay kit (Thermo Scientific, Waltham, MA, USA) according to the manufacturer’s recommendations.

### 2.12. HPLC-MS/MS Data Acquisition and Analysis

Proteomic analysis of peptide mixtures was performed using the Ultimate 3000 RSLCnano (Thermo Scientific, USA) system equipped with a Q-Exactive HFX (Thermo Scientific) mass spectrometer. One microliter of the peptide mixture was applied to an Acclaim µ-Precolumn (0.5 × 3 mm, 5 μm) at a flow rate of 10 μL/min for 4 min under isocratic mode using buffer C as a mobile phase (2% acetonitrile, 0.1% formic acid in deionized water). Then, the peptides were separated using the Acclaim Pepmap^TM^ C18 (75 μm × 150 mm, 2 μm) (Thermo Scientific) column under the following gradient of B (80% acetonitrile in 0.1% aqueous formic acid) in A (0.1% aqueous formic acid): 2% for 10 min, from 2 to 35% for 68 min; from 35 to 99% for 2 min; 99% for 2 min; from 99 to 2% for 3 min. Total duration of the analysis was 90 min.

Mass spectrometry analysis was performed under positive ionization mode using the NESI source (Thermo Scientific). The following settings were used: emitter voltage 2.1 kV; capillary temperature 240 °C. Panoramic scanning was performed in the mass range from 300 to 1500 *m*/*z* at 120,000 resolution. For tandem scanning, 15,000 resolution in the mass range from 100 to the upper limit was determined automatically from precursor mass, but not more than 2000 *m*/*z* was used. Precursor ions were isolated in the ±1 Da window. The maximum number of resolved ions under the MS2 mode was set as not more than 40, the limit of precursor choice for tandem analysis was set at 50,000 units, and normalized collision energy (NCE) was 29. For tandem scanning, only ions with z form 2+ to 6+ were considered. The maximum time for precursor ion accumulation was 50 ms, and for fragment ions, 110 ms. The AGC (Automatic Gain Control) value for precursor and fragment ions was 1 × 10^6^ and 2 × 10^5,^ respectively. All measured precursors were dynamically excluded from tandem MS/MS analysis for 90 s.

Raw MS data files were analyzed using the MaxQuant v. 2.1.0.0 software with the Andromeda searching algorithm [59]. The UniProt FASTA human protein database (April 2022) was used for identification. The following search parameters were used: cleaving enzyme, trypsin; two missed cleavages allowed; monoisotopic peptide mass determination precision, ±4.5 ppm; mass determination precision in MS/MS spectra, ±20 ppm. Oxidation of methionines and acetylation at the N terminus was considered possible, and cysteine carbamidomethylation was a necessary modification. For validation of Peptide-Spectrum Matches, FDR (False Discovery Rate) < 0.1% was used for peptide and protein identifications. Proteins were considered reliably identified if at least two peptides were detected. LFQ (label-free quantification) and iBAQ (intensity-based absolute quantification) values were used to evaluate protein quantities.

In order to evaluate the content of the proteins in each experimental group of samples, the relative protein abundance, RPA (%), was calculated based on the iBAQ values. For this, the sum of the iBAQ values in the technical replicates for each protein was divided by the sum of iBAQ values for all identified proteins in the sample and multiplied by 100%. Statistical analysis of proteomic data was carried out using the Perseus v.1.6.15.0 software. In order to compare proteins between samples, the protein iBAQ (to calculate relative protein abundance or RPA %) or LFQ (to compare protein intensities between samples) values of the samples were loaded into the program, including technical replicates. Four groups were formed: control plasma, MLPH, 5SX, and 10SX (three or four replicates for two independent samples each). The data were filtered out beforehand: possible contaminant proteins and false positive identifications were removed; proteins identified by two or more peptides and present in at least 70% of samples in at least one isolated group were left for analysis. Then, data were log2-transformed and normalized using the z-score function. Missing values were replaced from the normal distribution. In order to determine statistically significant differences between the groups, the multi-sample ANOVA with post-hoc Tuckey’s HSD test (FDR 0.05) was used. Statistically significant differences were considered with a fold change > 2 and a value of q < 0.01.

### 2.13. Delipidization of Liposome–Protein Complexes, SDS-PAGE, and Immunoblotting

Delipidization was performed as described in [60] and in our recent study [52]. The resulting protein samples were dissolved in 36 μL of 2× standard reducing buffer for SDS-PAGE and boiled 2 × 2 min. SDS-PAGE was carried out in 6% concentrating and 12% separating gels on a Mini Gel Tank (Thermo Fisher Scientific, Waltham, MA, USA) setup for 47 min at 200 V. Precision Plus Protein^TM^Dual Color Standards (Bio-Rad Laboratories, Inc., Hercules, CA, USA) was used as molecular weight marker. Proteins were visualized by silver staining or transferred to a PVDF membrane using the Mini Gel Tank (Thermo Fisher Scientific, Waltham, MA, USA) at 20 V for 60 min. After the end of the transfer, the membrane was washed with TBS and, to prevent non-specific sorption, incubated in 5% low-fat dry milk in TBS with 0.1% Tween 20 (TBS/T) for 1 h at room temperature. Then, the membrane was washed with TBS/T (3 × 5 min) and incubated with primary antibodies (anti-IgG, IgM, C3, C4BP, fI, Vne, HSA, ApoA1, ApoE, ApoH) in 0.5% BSA solution for 2 h at room temperature. After that, the membrane was washed with TBS/T (15 min and 3 × 5 min), incubated with secondary antibodies conjugated with horse-radish peroxidase at 4 °C overnight, and then washed again with TBS/T 5 × 5 min. Immunodetection was performed with Clarity™ ECL Western Blotting Substrate (Bio-Rad) reagent and a VersaDoc 4000 (Bio-Rad) system.

### 2.14. Statistics

For each liposome sample, incubation and further treatment were performed thrice. The relevant data obtained in experiments under static conditions are presented as means ± SD. Specific statistical tests and software used in microfluidic chip experiments and in proteome analysis are described in Section 2.8 and Section 2.12, respectively.

## 3. Results and Discussion

### 3.1. Characteristics of Liposomes

Liposomes loaded with the melphalan lipophilic prodrug, MlphDG, are formed on the basis of natural phospholipids egg phosphatidylcholine and soybean phosphatidylinositol, which provide for a fluid-phase bilayer. The composition allows for avoiding heating during liposome preparation, which accelerates the degradation of alkylating groups of the melphalan module. Fluid-phase bilayer can also facilitate the intracellular unloading of liposomes. The composition, measured size, and zeta-potential values of the liposomes used in this study are presented in Table 1.

**Table 1:** Composition and physicochemical characteristics of the liposome samples.

Sample Name	Liposome Composition, Mol Ratio	D, nm ^1^ Average ± SD	PdI ^1^, Average ± SD	Zeta-Potential, mV ^2^ Average ± SD	
				Liposomes	Liposome–Protein Complex
ePC	ePC	120.0 ± 1.1	0.028 ± 0.020	−3.3 ± 0.4	N/A
MLPH	ePC–PI–MlphDG, 8:1:1	130.2 ± 1.4	0.087 ± 0.044	−8.8 ± 0.8	−11.7 ± 0.5
5SX	ePC–PI–MlphDG–SiaLe^X^, 7.5:1:1:0.5	122.7 ± 0.8	0.069 ± 0.025	−25.0 ± 0.9	−22.1 ± 0.5
10SX	ePC–PI–MlphDG–SiaLe^X^, 7:1:1:1	102.7 ± 1.0	0.079 ± 0.042	−31.7 ± 1.5	−28.1 ± 0.6

^1^ As assessed using Brookhaven Particle Analyzer 90+ (Brookhaven Instruments Corp., Holtsville, NY, USA); PdI, polydispersity index. ^2^ Data for ~200-nm liposomes; zeta-potentials were measured using Litesizer 500 (Anton Paar GmbH, Graz, Austria).

When equimolar amounts of negatively charged PI and MlphDG with a protonated primary amino group (pH 7.4, PBS) were included in practically neutral ePC liposomes composed of zwitterionic phosphatidylcholine molecules, PI not only compensated for the MlphDG charge but also shifted the electrokinetic potential of the liposomes to a more negative value (−8.8 mV). Such a result can be explained by the structural nonequivalence of the PI and MlphDG polar head groups and the different arrangement of their charged groups on the surface of the lipid bilayer, where phosphates protrude into the aqueous phase somewhat more than protonated amino groups. The inclusion of increasing amounts of SiaLe^X^ conjugate exposing the dissociated carboxyl group in the MLPH liposome bilayer led to a further progressive increase in the negative charge of the liposomes (from −25 to −31.7 mV). When liposomes were incubated with 50% human plasma and liposome–protein complexes were separated from free proteins, we observed slight changes in liposome zeta-potentials (Table 1). Adsorbed proteins have moderately shifted all values closer to the common value of −20 mV. This value is typical of liposomes, and nanoparticles in general, covered with a layer of plasma proteins irrespectively of the initial nanoparticle surface charge, which is thought to be due to the fact that most plasma proteins carry net negative charge at physiological pH [61,62].

### 3.2. Hydrodynamic Conditions in Microfluidic Chips

The use of microfluidic technologies for the development and testing of targeted drug transport to cells and tissues is becoming a widespread practice [63,64]. In this study, microfluidic chips with four channels were designed (Figure 2a) to allow for repeated measurements within a single experiment, while the surface area in each channel was made large enough to ensure the formation of a monolayer of more than 10,000 HUVEC cells.

The magnitudes of shear stress vary in different parts of the vascular system. In the arteries, the flow is highly pulsating, and the shear stress is in the range of 1–5 Pa. A similar amplitude, although with less pulsation, is observed in the capillaries. In the veins, it is 0.1–0.5 Pa with minimum pulsation [65,66,67]. Similar values are reported for individual elements of the vascular bed in other sources: mean shear stress often is <0.1 Pa in the large veins; it can reach 6–8 Pa in small arterioles and 2–4 Pa in small venules [68,69].

The choice of hydrodynamic conditions for the microfluidic setup was made, taking into account the venous bed origin of HUVECs. Moreover, the monolayer of endothelial cells in the chip has less elastic mechanical compensation capabilities due to a lack of interaction with other natural elements of connective tissue. Hydrodynamic calculations showed that at a volume flow rate (hereinafter referred to as flow rate) of 100 μL/min, the shear stress in the channel reaches 0.2 Pa (Figure 2b). Considering the above, we used a non-pulsating constant unidirectional laminar flow with a flow rate of 100 μL/min as a physiological flow in the channels of the microchip.

### 3.3. Liposome Uptake by HUVEC Cells in Microfluidic Chips

The effectiveness of internalization by HUVEC cells of fluorescently labeled liposomes under dynamic conditions was studied depending on a number of factors: the presence/absence of the selectin ligand SiaLe^X^, activation by proinflammatory cytokine TNF-α, flow rate, and medium composition.

**Figure 3:**
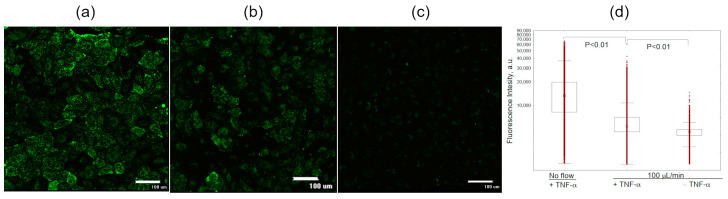
Monolayer of HUVEC cells internalizing 5SX liposomes. Images of microchannel fragments with HUVEC cells after activation for 4 h with 50 ng/mL TNF-α: (**a**) under static conditions, (**b**) under flow of 100 µL/min, or (**c**) under flow, without the activation. (**d**) Distribution of fluorescence intensities of HUVEC cells internalizing 5SX liposomes; data on internalization under static conditions (NO FLOW) or with a flow rate of 100 µL/min. Data are presented as medians, 25–75 percentiles, and ranges of values without outliers. Kruskal–Wallis criterion and the median test were used to compare the groups. The monolayer of cells seeded into the microchannels for 18 h was activated for 4 h with 50 ng/mL TNF-α. Control cells were not activated (–TNF-α). Then, the cells in the chip were exposed to a flow of liposome suspension in Fluorobrite with 20% FBS for 15 min.

Activation with TNF-α increased the accumulation of liposomes carrying 5% SiaLe^X^ ligand in cells under the flow of the medium supplemented with 20% FBS (Figure 3). Under static conditions, the internalization of 5SX liposomes by activated cells was significantly higher than under flow conditions (Figure 3c). Apparently, when the flow velocity at the wall is nonzero, the time of contact between the ligand and the receptor decreases and might not be enough for the formation of a bond.

**Figure 4:**
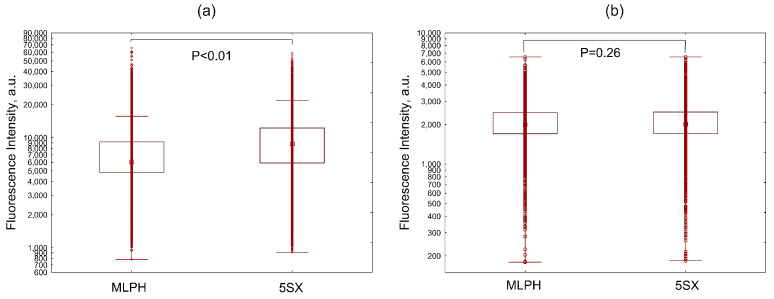
Distribution of fluorescence intensities of HUVEC cells internalizing liposomes without a ligand (MLPH) or 5SX liposomes after activation with TNF-α (**a**) or without activation (**b**) in 20% FBS under a flow rate of 100 µL/min. Data are presented as medians, 25–75 percentiles, and ranges of values without outliers. Kruskal–Wallis criterion and the median test were used to compare the groups.

Under the same conditions, the 5SX liposomes were internalized by activated cells more efficiently than the MLPH ones (Figure 4a). In the absence of activation, there was no difference in the uptake of the two types of liposomes (Figure 4b). This evidences the contribution of receptor-mediated endocytosis during the internalization of SiaLe^X^-liposomes by activated HUVEC cells under flow conditions.

**Figure 5:**
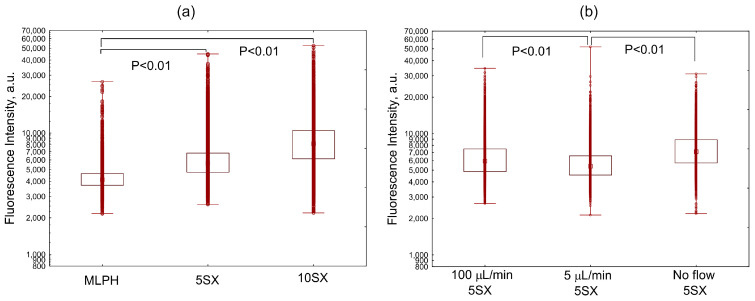
Comparison of distributions of fluorescence intensities of activated HUVEC cells internalizing: (**a**) liposomes with various content of the SiaLe^X^ ligand: MLPH vs. 5SX and 10SX samples min at a flow rate of 100 µL/min, and (**b**) 5SX liposomes at a flow rate of 0, 5, and 100 µL/min. A monolayer of cells pre-seeded into the microchannels for 18 h was activated for 4 h with 50 ng/mL TNF-α, then exposed to the flow of a suspension of liposomes in the blood serum of healthy donors for 15 min. Data are presented as medians, 25–75 percentiles, and ranges of values without outliers. Kruskal–Wallis criterion and the median test were used to compare the groups.

Under flow conditions in human serum, the efficiency of internalization of 5SX liposomes was higher than that of the SiaLeX-free ones, although the level of liposome consumption decreased compared to the experiment in the growth medium (compare Figure 4a and Figure 5a). We assume that liposome internalization by cells is mediated by the protein corona formed on the liposome surface in both FBS and human serum. Competition for the receptors is higher in human serum due to the higher total protein concentration. This could explain lower liposome uptake by cells in human serum, compared to 20% FBS.

The 10SX liposomes were almost twice more efficiently captured by activated cells in a flow of 100 µL/min compared to the liposomes without a ligand (Figure 5a). These results support the involvement of specific interactions between SiaLe^X^-liposomes and activated endothelial cells under the conditions of a dynamic flow of human serum and could be translated to the situation in the blood vessels, although without taking into account the participation of blood cells.

The flow of growth medium with 20% serum reduced the internalization of SiaLe^X^-liposomes by activated cells in comparison with static conditions (Figure 3c). A similar trend, although statistically not significant, was observed in human serum (100 µL/min vs. 0 µL/min; Figure 5b). Presumably, higher competition with the more abundant proteins in human serum (compared to 20% FBS) leads to overall lower liposome uptake by the cells. Against this low uptake, the difference between flow and quasi-static conditions is less pronounced.

**Figure 6:**
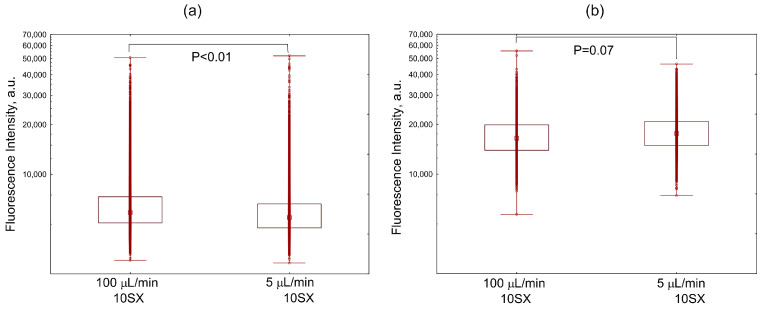
Distribution of fluorescence intensities of activated HUVEC cells internalizing the 10SX liposomes under flow (100 µL/min) and quasi-static (5 µL/min) conditions for 15 min in the blood serum (**a**) or in the Fluorobrite growth medium without serum (**b**). See Figure 5 caption for cell preparation conditions and data presentation details.

Unexpectedly, the uptake of SiaLe^X^-loaded liposomes into activated cells was more efficient under human serum flow of 100 µL/min than under quasi-static conditions (5 µL/min), although the difference was weakly expressed (Figure 5b). Cell accumulation of 10SX under quasi-static conditions (5 µL/min) also was slightly, yet significantly, lower than under flow conditions (100 µL/min, Figure 6a). There was no difference between flow and quasi-static conditions in a medium without serum (Figure 6b), but the intensity of cell fluorescence exceeded that acquired in serum (Figure 6a).

In general, our results suggest that the protein corona can reduce the effectiveness of receptor-mediated endocytosis in the flow and decrease the absorption of SiaLe^X^-liposomes under serum conditions. Among the additional factors that may influence the cellular uptake of SiaLe^X^-containing liposomes, the effect of the shear threshold could be noted. In the flow, carriers modified by ligands with low affinities, such as SiaLe^X^ or SiaLe^A^, are thought to bind cell surface through “rolling” and not by strong adhesion, with lower shear deformations being accompanied by low adhesion; thus, maximum binding occurs at intermediate values of the flow velocity [70,71,72]. Apparently, with different densities of the protein corona and, accordingly, different availability of the low-affinity targeting ligand for interaction with the receptor molecule, the intermediate values of the flow velocity accompanying the shear threshold are to be different. Thus, the same flow rates can differently affect the particles, carrying different (due to the different density of shielding protein corona) amounts of ligand available to the receptor, with respect to their interactions with target cells.

### 3.4. Liposome Uptake by Activated HUVECs under Static Conditions

**Figure 7:**
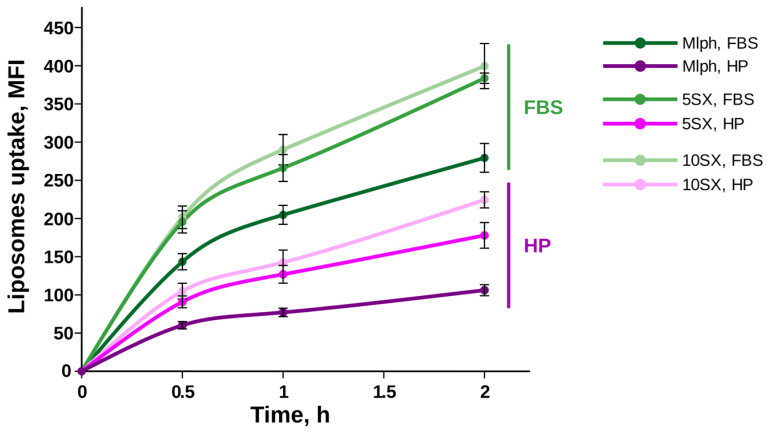
Liposome uptake by activated HUVECs under static conditions. Liposomes (100 µM total lipids) with protein corona formed in FBS or human plasma (HP) were incubated with cells and analyzed by flow cytometry. Representative experiment, mean fluorescence intensity (MFI) ± SE.

To test the effect of the protein corona on liposome–cell interactions under more simple conditions, independent of the complex influence of flow rates and shear stress, we conducted experiments under statics. When liposome uptake by activated HUVECs was studied under static conditions without a microfluidic chip, the results were similar to those discussed above. The level of consumption of liposomes incubated in human plasma was yet again lower than that after incubation in FBS (Figure 7). Yang and co-authors noted that not only the higher quantity of proteins in human serum perplexes liposome endocytosis, but also free proteins from human serum can have higher affinity to cell receptors on the human cells used in experiments than bovine serum proteins [73].

Previously, we observed an increased accumulation of SiaLe^X^ liposomes by HUVECs under serum-free conditions, with the growth of the SiaLe^X^ content in the bilayer [32]. Here, we show that increasing the amount of SiaLe^X^ facilitated liposome absorption by endothelial cells in the presence of serum proteins independently of the source of the proteins (see Figure S1 for combined data).

### 3.5. Plasma Protein Binding by Liposomes: General Results of the Proteome Analysis

Since we observed a potential interference of the protein corona in the ligand-mediated uptake of the MlphDG liposomal formulation decorated with SiaLe^X^ by endothelial cells, we analyzed the proteome of liposome–protein complexes formed in human blood plasma. The best option would have been to compare the protein coronas formed under flow conditions within the microfluidic chips that we used. However, the experimental setup dramatically limited sample volume and, consequently, protein content in the samples of liposome–protein complexes. Therefore, we explored the protein coronas formed under static conditions.

To separate liposome–protein complexes from plasma, we relied on an SEC procedure, taking into account considerations of Kristensen and co-authors regarding possible contaminations with plasma proteins [74]. Namely, we centrifuged pooled EDTA human plasma prior to incubation with liposomes to get rid of contaminating protein aggregates. We did use ultrafiltration to concentrate liposome-containing fractions prior to protein analysis, being aware of the possibility of lower protein recovery in the absence of liposomes. However, in contrast to the above-mentioned work [74], we only used a single 300,000 kDa filtration step with no extensive washings. We estimated the protein content in blank plasma samples in the liposome-corresponding fractions to be roughly a third of the protein content in the peaks of the liposome-containing samples. For this reason, for LC-MS/MS and Western blotting experiments, each plasma control sample was prepared by joining three SEC runs. We then confirmed that there were no significant differences in the peptide concentrations between liposome and control plasma samples assayed prior to the LC-MS/MS analysis (see Figure S3). Thus, we consider the blank plasma sample a valid control for contaminants.

**Figure 8:**
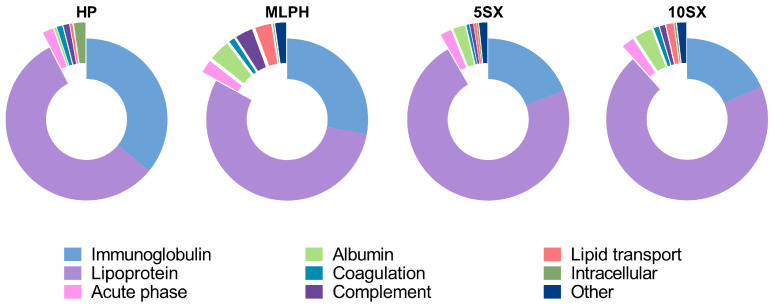
Average distribution of liposome-associated proteins over major functional clusters for proteins with RPA > 0.1%. See Supplementary Table S1 for the whole list of proteins and iBAQ values.

According to LC-MS/MS, the control plasma sample consisted mainly of apolipoproteins and immunoglobulin fragments. These could contribute to the composition of liposomal coronas as contaminants. However, some of these proteins were depleted from the liposomal samples (e.g., apolipoproteins E and A4) compared to the plasma control according to LFQ intensities. All studied liposome protein coronas were also depleted from von Willebrand factor (reported to belong to top-10 plasma contaminants upon SEC separation by Kristensen and co-authors, together with immunoglobulin mu chain, apolipoprotein B48/100, and several intracellular proteins), IgGFc-binding protein, galectin-3-binding protein, and CD5L. They were enriched with albumin, apolipoproteins F and M, cathelicidin, and ficolin-2. Apolipoprotein C1, serum amyloid A4 (SAA4) protein, and immunoglobulin heavy constant gamma 1 chain were enriched on SiaLe^X^-containing liposomes. Proteins associated with the MLPH liposomes were more functionally diverse compared to other samples (see Figure 8 for relative protein contents in the samples and Supplementary Table S2 for the results of the intersample comparison of LFQ intensities).

Albumin was only observed to be associated with the liposomes (and not in blank plasma samples), which speaks for its accumulation on the surface thereof. Relative protein abundances (RPAs) of albumin, immunoglobulins, and complement proteins were the highest for the MLPH sample compared to 5SX and 10SX. The contribution of total lipoproteins gradually increases in the row HP < MLPH < 5X < 10SX. Also, within the lipoprotein group, contributions of various apolipoproteins changed from one liposome sample to another. Particularly, apolipoprotein C2, a component of VLDL (very low-density lipoproteins) and chylomicrons, contributed roughly 20–30% of the protein in all liposome samples (Table 2). Since it is also the most abundant protein in plasma fractions eluted from the same column as liposomes (27.6 ± 5.4%; no significant differences in LFQ intensities between 5SX/10SX and plasma control), one can suggest VLDL and chylomicrons are eluted together with liposomes as contaminants.

As for the growth of the lipoprotein fraction in samples 5SX and 10SX, this may be attributed to the accumulation of the positively charged ApoC1 in the protein coronas of liposomes decorated with polyanionic SiaLe^X^ ligands on their surface. The RPA of ApoC1 in the corona of 5SX liposomes increased approximately two times in comparison with the SiaLe^X^-free sample and continued to increase in liposomes 10SX. According to the LFQ intensities, ApoC1 was significantly enriched on all liposomes, 5SX and 10SX were also enriched compared to MLPH, and 10SX was enriched versus 5SX. ApoC1, the smallest protein of all known apolipoproteins, 6.7 kDa, with a normal plasma concentration of 0.04–0.07 mg/mL, is one of the most positively charged proteins of the human body (pI 8.3) [75]. It is associated with both HDL (high-density lipoproteins) and VLDL. It is possible that ApoC1 contributes most to the shielding of SiaLe^X^ ligand from specific interactions with E-selectin on activated endothelial cells, which we observed in the human serum flow in a microfluidic device. Liposomes are characterized by lower content of ApoE compared to blank plasma, which indicates VLDLs are more likely contaminants of liposome–protein complexes isolated by SEC rather than particles interacting with the liposomes. Notably, according to the LFQ intensities data, liposome protein coronas were enriched with apolipoprotein M, a component of HDLs. Some of the protein constituents of HDLs were enriched only in selected liposomal samples, particularly apolipoprotein C1 and serum amyloid A4 (SAA4) protein on 5SX and 10SX and apolipoprotein C4 on 10SX. These data suggest that HDL components are selectively adsorbed on liposome surfaces. SAA4, constitutively expressed in the liver and secreted into plasma (~0.055 mg/mL) as part of HDLs, serves as a precursor to the fibrillar tissue protein AA. The content of SAA4 increases rapidly in the acute phase of inflammation in patients with venous thrombosis [76]. By taking into account the connection of selectin expression with inflammatory processes, it can be assumed that their ligand—SiaLe^X^—is somehow indirectly involved in interaction with apolipoprotein SAA4.

Literature data on the protein corona proteome of anionic liposomes are scarce. An early work by Caracciolo and co-authors suggests lower content of total apolipoproteins (below 20%) and immunoglobulins (below 15%) for 100% POPG liposomes [77]. The authors used low-speed centrifugation (14,000× *g*, 15 min) to isolate liposome–protein corona complexes. While the technique could hardly result in the sedimentation of lipoproteins, liposome–protein complexes also should have been isolated incompletely. This explains the difference in the corona compositions with samples in the current study, in addition to lipid composition-specific differences.

### 3.6. Plasma Protein Binding by Liposomes: Selected proteins

Along with the data of proteomic analysis, Western blotting (WB) provides verification and refinement of data on the binding of individual proteins to liposomes.

**Figure 9:**
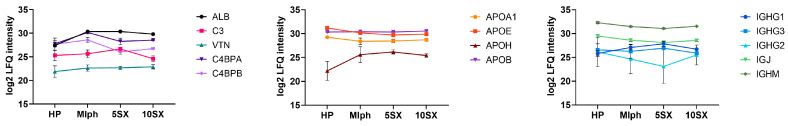
Binding profiles (log2 LFQ intensities) of selected plasma proteins according to LC-MS/MS data. log2 LFQ intensities averaged (mean ± SD) over all replicates (n = 7 for HP and 6 for other samples). See Supplementary Table S1 for the whole list of proteins and log2 LFQ intensity values.

**Figure 10:**
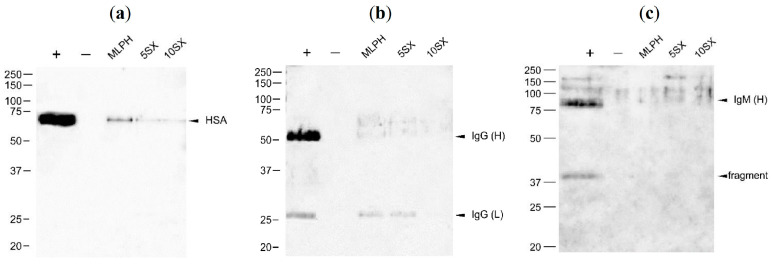
Western blots of liposomal protein corona components with anti-HSA (**a**), anti-IgG (**b**), and anti-IgM (**c**) antibodies. +, positive control, human plasma diluted 1250-fold; −, negative control, buffer and plasma mix treated the same way as the liposomes. IgG is presented by heavy and light chain bands, and IgM is presented by a heavy chain and its fragment due to SDS-PAGE-reducing conditions.

*Albumin* is the most abundant serum protein, with a concentration of 35–50 mg/mL [78]. Human serum albumin (HSA) adsorption on the surface of liposomes can have ambiguous outcomes: it can both prolong liposome circulation time [79] and induce macrophage clearance and complement system activation [80,81]. Fleischer and co-authors [82] have shown that if the protein binding to the nanoparticle surface requires/induces changes in HSA secondary structure, the modified structure is recognized by a scavenger receptor SR-B1. All liposome samples were significantly (*p* < 0.01) enriched with albumin compared to the plasma control sample according to LFQ intensities obtained in the LC-MS/MS analysis (Figure 9). Moreover, according to WB (Figure 10a), the following relationship of HSA content in liposome-associated samples can be established: MLPH > 5SX > 10SX > plasma control.

The MLPH sample had the most prominent HSA band in WB, excluding diluted plasma control (Figure 10). Previously we observed BSA binding to MLPH liposomes, but there were no prominent changes in protein structure due to the presence of PI molecules in the bilayer [37]. The addition of the SiaLe^X^ conjugate decreased albumin adsorption onto liposomes (Figure 10a).

*Immunoglobulins* are other major components of human plasma. In this study, we were mostly interested in two classes, IgG and IgM, as they can initiate activation of the classical pathway of complement and be recognized by macrophages when adsorbed onto liposomes. Their plasma levels are 13.5 (IgG) and 1.5 mg/mL (IgM) [83]. Figure 10b shows low IgG binding to the MLPH sample that becomes even less prominent upon the SiaLe^X^ conjugate addition to the bilayer. IgM band intensity for liposomes is comparable to the signal in the negative control lane (Figure 10c). According to LC-MS/MS data, IgG heavy chain gamma fragments 1 and 3 were enriched on 5SX over 10SX sample; IgM content was higher on 10SX liposomes compared to MLPH; otherwise, there was no significant difference between liposomal samples in terms of IgG and IgM adsorption.

Due to their large size, IgM pentamers can be concurrently eluted with the liposomes in addition to liposome binding. The low amount of IgM detected in the WB most probably reflects the low sensitivity of the combination of primary and secondary antibodies used (in an independent run lacking a negative control due to the limited volume of the blank plasma sample, significant IgM is observed associated with all liposome samples; see Figure S4).

**Figure 11:**
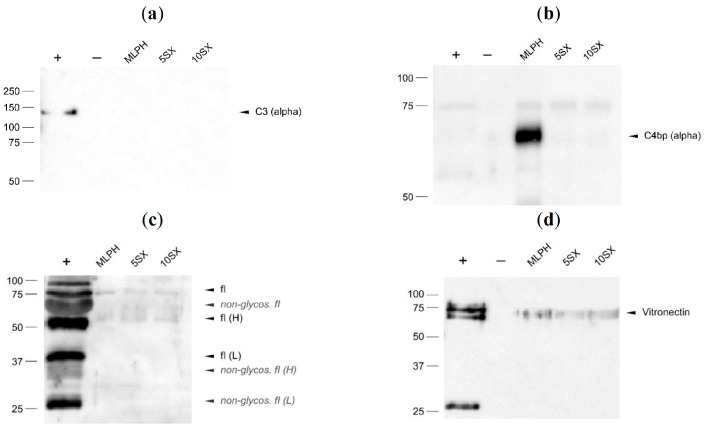
Western blots of liposomal protein corona components with anti-C3 (**a**), anti-C4BP (**b**), anti-fI (**c**), and anti-vitronectin antibodies (**d**). +, positive control, human plasma diluted 1250-fold (**a**,**b**,**d**) and 100-fold (**c**); −, negative control, buffer and plasma mix treated the same way as the liposomes. C4b-binding protein and factor I were partially reduced to various chains during SDS-PAGE sample preparations. The factor I fragments were assigned according to [86].

*Factor C3* is the most abundant protein of the complement system (1.2 mg/mL) [84]. It plays a central role in the complement cascade development as it is involved in all three activation pathways [85]. Classical, alternative, and lectin pathways lead to C3 conversion to anaphylatoxin C3a and fragment C3b, which binds to foreign surfaces triggering the amplification of complement cascade reactions. On the surface of the liposomes, we did not detect either C3 or its cleavage fragments (Figure 11a). According to z-score normalized log2 LFQ intensities, the 5SX sample was significantly enriched with C3 compared to 10SX, MLPH, and control plasma. Yet, in agreement with the WB data, C3 was not found among the top-10 proteins by the RPA values in the coronas of all liposomes (Table 2), its content exceeding 0.1% only in the case of the 5SX sample.

When the classical or lectin complement pathway is activated, it can be inhibited by *C4b-binding protein (C4BP)*. C4BP is a major effector protein in the complement cascade with plasma levels of ~0.25 mg/mL. This protein consists of 7 identical α-subunits (75 kDa) and 1 β-subunit (45 kDa), with an overall molar mass of about 500 kDa [87]. C4b-Binding protein concentrated the most on MLPH liposomes without a targeting ligand (Figure 11b). The same profile is observed in the LC-MS/MS intensities (Figure 9). According to z-score normalized log2 intensities, MLPH liposome corona was significantly enriched with C4BP subunits A (α) and B (β) compared to samples 5SX, 10SX, and control plasma. C4BP has a flower-shaped structure with each α-chain and unique β-chain radiating out from a central core, where they are linked by disulfide bonds. The β-chain-containing C4BP in circulation is bound to vitamin K-dependent anticoagulant protein S (PS), forming the C4BP–PS complex [87]. The n-Terminal γ-carboxyglutamic acid-rich domain of PS binds negatively charged phospholipids exposed by activated endotheliocytes (or by platelets during coagulation) through the complexation of Ca^2+^ cations [88]. Due to such structural and functional properties of C4BP, its increased binding by MLPH liposomes can be explained by their negatively charged surface; in the case of SiaLe^X^-liposomes, though even more negatively charged, it is sterically difficult for a flower-like large complex of C4BP–PS to be accommodated on their surface.

Another complement inhibitor, *factor I*, a ∼88 kDa heterodimer serine protease, cleaves C3b and C4b (free or bound) in the presence of C4BP or factor H as a cofactor [89]. Its average concentration in plasma is relatively similar to that of vitronectin (see below), 0.04 mg/mL [90]. Factor I was also detected on our liposomes by WB (Figure 11c). The addition of SiaLe^X^ does not appear to have affected factor I binding. Yet, factor I was not found among differentially adsorbing proteins in liposome samples upon LC-MS/MS, nor did it have RPA >0.1% in any of the samples (Table 2).

*Vitronectin* is a 75-kDa glycoprotein and yet another complement-regulating protein that is bound to the liposome samples according to WB (Figure 11d). According to LC-MS/MS data, low vitronectin binding was typical of all studied samples. Vitronectin inhibits complement cascade at later stages than C4BP and factor I. It binds membrane attack complex (C5b-7) and polymerization of C9, and formation of a pore in the lipid bilayer, which would cause pathogen cell lysis. Its plasma level is low, at 0.034 mg/mL [85]. Palchetti and others reported that vitronectin could be a targeting ligand on the surface of liposomes due to its recognition by ανβ3 integrins overexpressing tumor cells [91,92,93]. Both factor I and vitronectin are minor components of the protein corona, and their adsorption does not differ between the liposome samples.

All in all, none of the studied liposomes should cause adverse reactions related to complement system activation due to the binding of several cascade inhibitors and the absence of C3 adsorption and cleavage. This agrees with the results of hemocompatibility testing, where none of the melphalan prodrug-containing liposomes, including the SiaLe^X^-targeted compositions, caused activation of complement [39].

*Apolipoprotein A1* (ApoA1, 28.3 kDa) is the main component of HDLs that specialize in excessive cholesterol transfer to the liver. Binding to lipoproteins is thermodynamically preferable for ApoA1, but it also circulates as a free protein in plasma. ApoA1 level in plasma is similar to that of C3 and is about 1–1.5 mg/mL, which makes it one of the most abundant plasma proteins [94].

**Figure 12:**
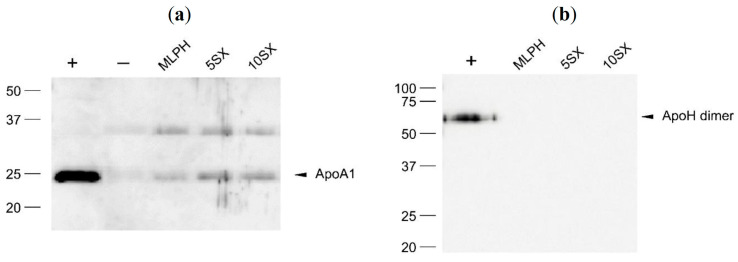
Western blots of liposomal protein corona components with anti-ApoA1 (**a**) and anti-ApoH (**b**) antibodies. +, positive control, human plasma diluted 1250-fold; −, negative control, buffer and plasma mix treated the same way as the liposomes.

In the blot obtained with anti-ApoA1 antibodies (Figure 12a), there is a specific ApoA1 band at 25 kDa and a not specific one, a bit lower than 37 kDa for all liposomes samples. We believe that the latter can be due to primary antibody cross-reactivity to ApoE (34 kDa). *Apolipoprotein E* (ApoE, 34 kDa) is a part of several classes of lipoproteins; it is involved in lipid metabolism throughout the body, including brain and nerve cells. Its plasma concentration is identical to that of factor I, 0.04 mg/mL, which is more than 25 times less abundant than ApoA1. Nonetheless, ApoE is a rather frequent component of the liposome protein coronas [41]. Researchers attempt to utilize ApoA1 and ApoE recognition by brain cells as a way for targeted delivery across BBB [95].

It should be noted that statistical analysis of LFQ intensities in the LC-MS/MS samples, which is more appropriate for intersample comparisons than RPA values, as well as WB data, shows that ApoE concentrations in liposomal samples were significantly lower than those in the plasma blank, in contrast to the ApoC2 and ApoC3, for which no differences with control plasma was noted. This favors the hypothesis that ApoC2 and ApoC3 are present within the analyzed sample due to having been eluted together with liposomes as part of VLDLs and chylomicrons. ApoE is probably eluted as a part of HDLs which normally can be separated by SEC under conditions that we used [96], yet some of it either contaminates the liposome–protein corona samples or is part of the coronas.

*Apolipoprotein H*, *or β2-glycoprotein 1*, is a 38.3 kDa phospholipid-binding protein with a plasma concentration of 0.2 mg/mL. The main function of β2-glycoprotein 1 is to bind phosphatidylserine on the surface of activated platelets and apoptotic cells. ApoH dimers bound to phosphatidylserine are recognized by antiphospholipid antibodies, which cause complement activation and attract phagocytes [97,98]. WB showed that none of our samples accumulated ApoH (Figure 12b), and the positive control lane has the only band corresponding to the protein dimer. At the same time, liposomal corona should be enriched with ApoH compared to the plasma blank sample, according to LC-MS/MS data (Figure 9). Relative protein content according to iBAQ intensities obtained in the LC-MS/MS experiment is 0.1% for the MLPH and 5SX samples and much lower for the other two; ApoH absolute content in WB samples could have been below the limit of detection.

Taken together, using shotgun proteomics and WB, we detected four “protein corona fingerprint” proteins, namely ApoA1, ApoC2, vitronectin, and IgG (identified by heavy chain), which were shown to facilitate liposome interactions with tumor cells [91]. The data on apolipoproteins in studied samples of liposome-associated proteins indicate that the described SEC procedure, which was largely adopted from the study by Kristensen and co-workers [74], most likely allowed us to separate small HDL particles, while larger VLDL and chylomicrons are probably present in the analyzed samples as contaminants.

## 4. Conclusions

Modeling of shear stress applied to the endothelium in capillary blood flow showed that targeting of activated endotheliocytes by SiaLe^X^-decorated fluid-phase antitumor liposomes persists, although the effect is less pronounced than under static conditions. In the flow of human serum, the interactions between the ligand on liposomes and cells are further dampened. Protein corona contributes to the weakening of liposome–cell interactions both in dynamic flow conditions and in statics. Liposome–protein complexes formed in human plasma are enriched with apolipoproteins. The content of the latter gradually increases with the increase in SiaLe^X^ ligand content. Supposedly, apolipoproteins could shield the ligand from the interactions with cell receptors. While ApoC2 as the first or second most abundant protein in all liposome coronas could contribute to higher liposome consumption by cells, the chance that ApoC2 merely co-elutes with liposomes (as a component of VLDL or chylomicrons, which have sizes and densities close to our liposomes) in the course of corona isolation, is high. At the same time, we found an increase in the content of ApoC1 in the corona of liposomes in the row MLPH < 5SX < 10SX. We hypothesize that ApoC1, together with other positively charged proteins, such as SAA4, interacts with negatively charged SiaLe^X^ residues on the surface of liposomes, competing for interactions with selectins on endothelial cells. On the other hand, ApoA1, vitronectin, and IgG found in coronas of all samples were shown to facilitate liposome interactions with tumor cells [91]. The interactions involved in liposome internalization by tumor and endothelial cells surely might be different. However, it is probably the interplay between shielding of targeting ligands and protein corona components’ own interactions with cell membrane that ultimately determine the intensity of the uptake. Elucidation of the role of plasma proteins highly abundant in the protein coronas of the SiaLe^X^-bearing targeting liposomes, identified in this study, in competition assays in tumor cells versus cells in the bloodstream could provide new insights for selective targeting of anticancer liposomes and their possible off-target effects.

## Figures and Tables

**Table 2:** Relative protein abundances (RPA %) of top-10 proteins for liposome samples and plasma control, mean ± SD.

	Plasma Control		MLPH		5SX		10SX	
	Gene Name *	RPA %	Gene Name	RPA %	Gene Name	RPA %	Gene Name	RPA %
1	APOC4-APOC2 **	27.6 ± 5.4	APOC4-APOC2	20.0 ± 9.8	APOC1	27.3 ± 4.1	APOC1	30.4 ± 1.1
2	IGHM	13.3 ± 2.9	APOC1	14.0 ± 0.8	APOC4-APOC2	24.2 ± 3.0	APOC4-APOC2	25.8 ± 1.8
3	APOE	9.3 ± 0.6	IGHM	11.0 ± 2.4	APOC3	8.4 ± 2.3	IGHM	8.1 ± 0.6
4	APOC1	7.1 ± 2.4	APOC3	8.0 ± 2.2	IGHM	7.7 ± 1.7	APOC3	6.8 ± 0.7
5	IGKC	7.0 ± 3.0	IGKC	6.3 ± 0.5	IGKC	4.2 ± 0.8	IGKC	4.1 ± 1.3
6	APOC3	6.6 ± 1.9	APOE	5.5 ± 0.5	APOE	4.1 ± 0.4	APOE	4.1 ± 0.5
7	IGLL5; IGLC1	3.8 ± 1.3	ALB	4.1 ± 0.5	ALB	3.5 ± 0.5	ALB	2.4 ± 0.2
8	IGJ	3.3 ± 1.0	APOD	3.2 ± 0.5	SAA2-SAA4	1.8 ± 0.3	SAA2-SAA4	1.7 ± 0.2
9	CD5L	1.7 ± 0.5	IGJ	2.4 ± 0.7	APOA1	1.4 ± 0.2	APOA1	1.4 ± 0.3
10	APOA2	1.5 ± 0.9	IGLL5; IGLC1	2.4 ± 0.5	APOM	1.4 ± 0.3	IGJ	1.4 ± 0.2

Note: See Supplementary Table S1 for the whole list of proteins and iBAQ values. * We report gene names rather than protein names because the algorithm compares experimental spectra with calculated spectra of the peptides that could have been produced if known human proteome genes (the UniProt FASTA human protein database) had been translated and then processed under the conditions of the LC-MS/MS experiment. ** ApoC2 peptides are considered to be derived from the ApoC4-ApoC2 read-through.

## Data Availability

The data can be made available upon request.

## Supplementary Materials

The following supporting information can be downloaded at: https://www.mdpi.com/article/10.3390/pharmaceutics15061754/s1, Supplementary Table S1: iBAQ and LFQ intensity values used to compare protein intensities within and between samples; Supplementary Table S2: Intersample comparison of LFQ intensities; Supplementary Figure S1: Combined data on the uptake of SiaLe^X^ liposomes by HUVECs in serum-free conditions and in the presence of serum; Supplementary Figure S2: The examples of photomicrographs of microchannels with HUVEC cells to Figure 3; Supplementary Figure S3: Total peptides in samples of liposome–plasma protein complexes; Supplementary Figure S4: Western blot of liposomal protein corona components with anti-IgM antibodies.
