# Supplementary material for: Protein Corona Attenuates the Targeting of Antitumor Sialyl Lewis X-Decorated Liposomes to Vascular Endothelial Cells under Flow Conditions
Source: Pharmaceutics. 2023 Jun 16;15(6):1754. doi: 10.3390/pharmaceutics15061754 (PMC10303272; doi:10.3390/pharmaceutics15061754)
Supplement: Supplementary file 1 [file pharmaceutics-15-01754-s001.zip › Supplementary figures.pdf]

Supplementary Material

Protein Corona Attenuates the Targeting of Antitumor Sialyl Lewis X-Decorated Liposomes to Vascular Endothelial Cells under Flow Conditions

Natalia R. Onishchenko [1,†,‡], Alexey A. Moskovtsev [2,†], Maria K. Kobanenko [1], Daria S. Tretiakova [1], Anna S. Alekseeva [1], Dmitry V. Kolesov [2], Anna A. Mikryukova [2], Ivan A. Boldyrev [1], Marina R. Kapkaeva [3], Olga N. Shcheglovitova [3], Nicolai V. Bovin [1], Aslan A. Kubatiev [2], Olga V. Tikhonova [4] and Elena L. Vodovozova [1,*]

[1] Shemyakin–Ovchinnikov Institute of Bioorganic Chemistry, Russian Academy of Sciences, ul. Miklukho-Maklaya 16/10, 117997 Moscow, Russia
[2] Institute of General Pathology and Pathophysiology, Russian Academy of Sciences, ul. Baltiyskaya 8, 125315 Moscow, Russia
[3] N.F. Gamaleya National Research Center for Epidemiology and Microbiology, Ministry of Healthcare of the Russian Federation, ul. Gamaleya 18, 123098 Moscow, Russia
[4] Institute of Biomedical Chemistry, ul. Pogodinskaya 10, 119121 Moscow, Russia
* Correspondence: elvod.ibch@yandex.ru
† These authors contributed equally to this work.
‡ Current address: Center for Soft and Living Matter, Institute for Basic Science, UNIST-gil 50 bldg. 103, Ulsan 44919, Republic of Korea.

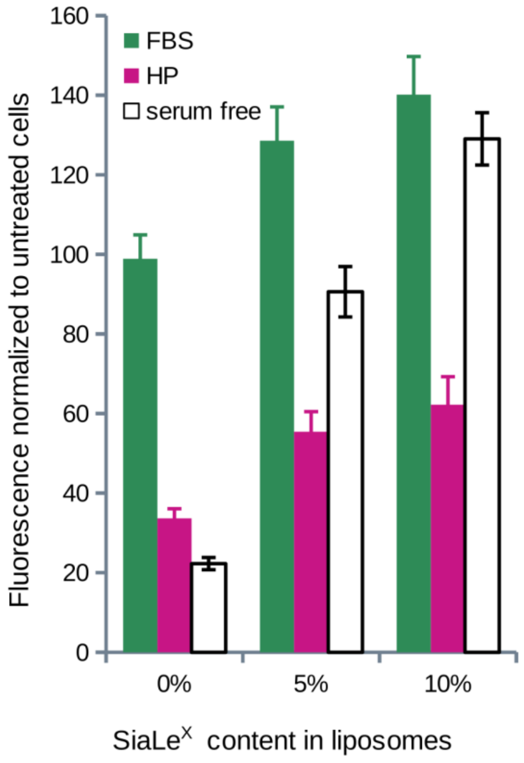

Figure S1. Accumulation of liposomes by HUVECs in function of the content of $SiaLe^X$ conjugate in liposomes in different conditions. Before addition to the cells, liposomes were pre-incubated (15 min, 37 °C) in serum free culture medium, FBS (90%) or human plasma (90%) to form protein corona. Then, liposomes were added to activated cells (TNF-α, 50 ng/mL, 4 h) and incubated for 1 h at 37 °C (100 μM total lipid in case of 10 times diluted 90% serum or plasma and 50 μM in serum free medium). Cells were detached with EDTA solution and analysed by FACS; data of a representative experiment are presented (mean±SE). Mean fluorescence intensity was normalized to the signal of control untreated cells. Data for serum free conditions were reproduced from our previous work 10.1016/j.bbamem.2015.01.016.

It is difficult to directly compare the results of these experiments, since different primary cultures of individual donor cells were used, as well as different concentrations of liposomes. Yet in all three variants of the experimental conditions, an increase in the amount of $SiaLe^X$ contributed to the absorption of liposomes by activated HUVECs. Another thing is that in the incubation medium without serum, the concentration of liposomes was two times lower, which may be the reason for higher cell uptake results after incubations with liposomes carrying a protein corona from FBS. However, in the case of a protein corona from human plasma, HUVECs did absorb a smaller amount of $SiaLe^X$-liposomes, despite their higher concentration than in a medium without serum.

NoFlow +TNF-α

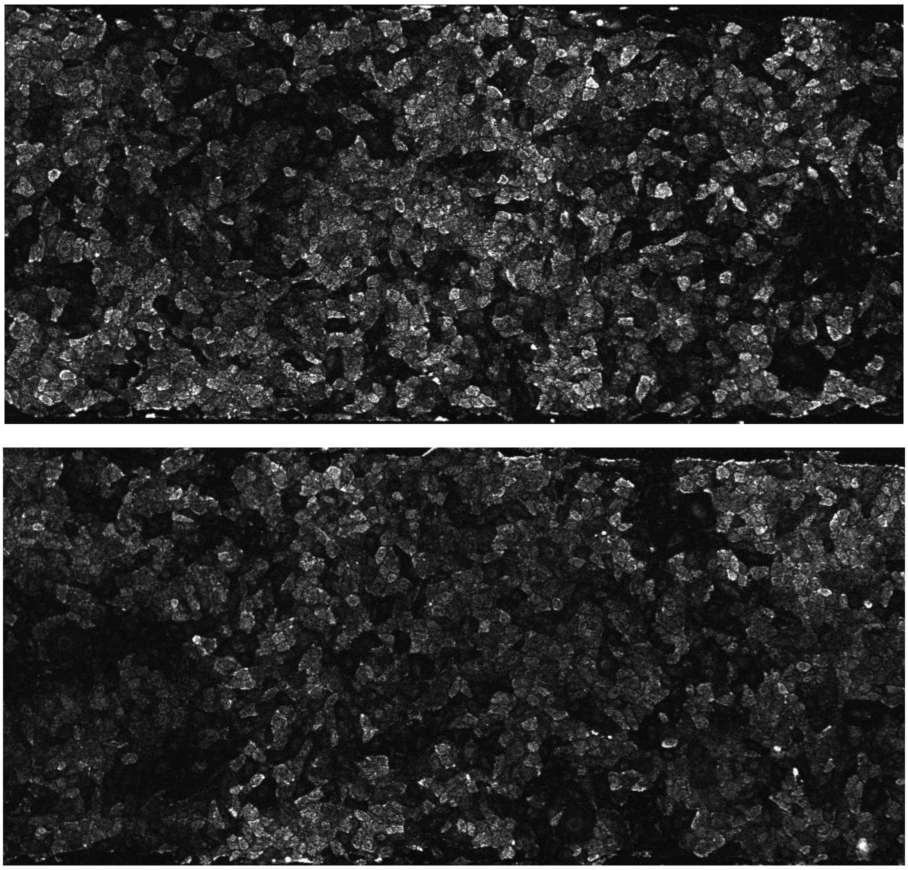

100 µL/min +TNF-α

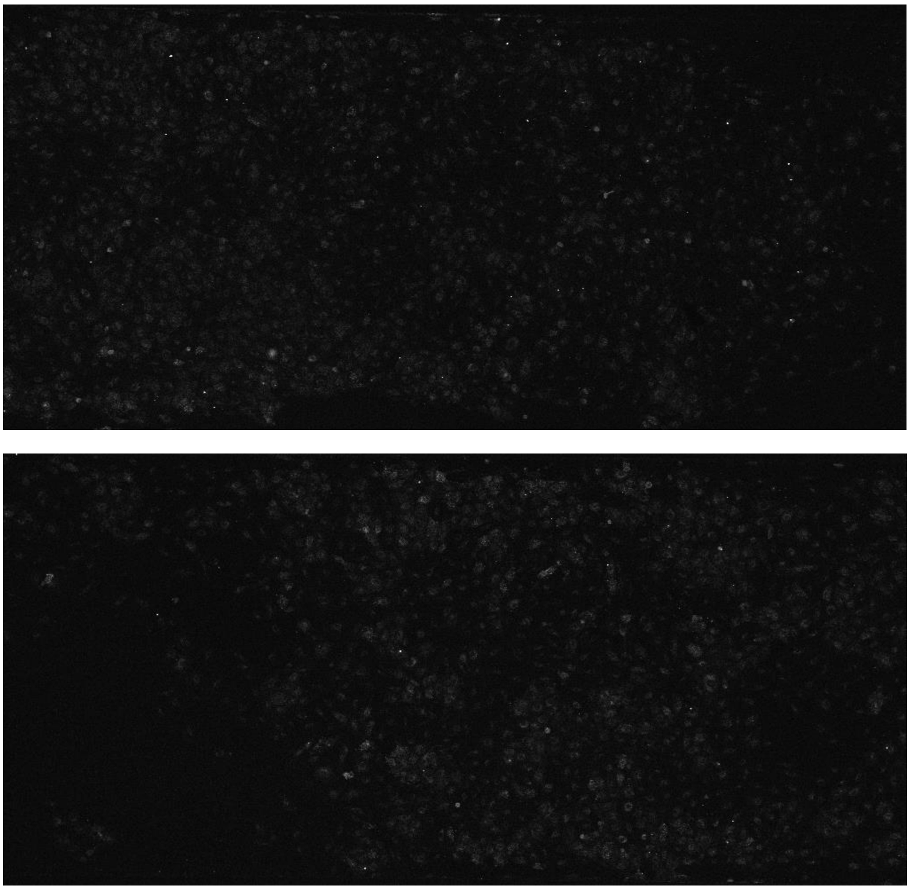

100 µL/min –TNF-α

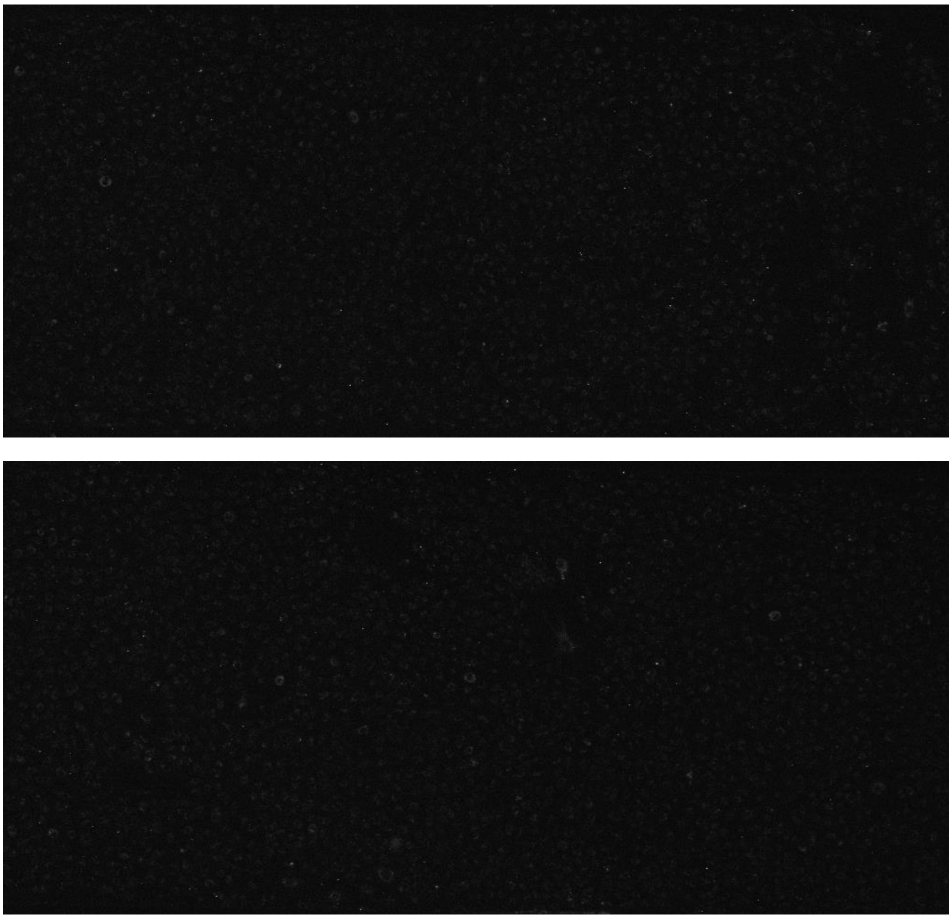

Figure S2. The examples of micrographs of microchannels with HUVEC cells to Figure 3.

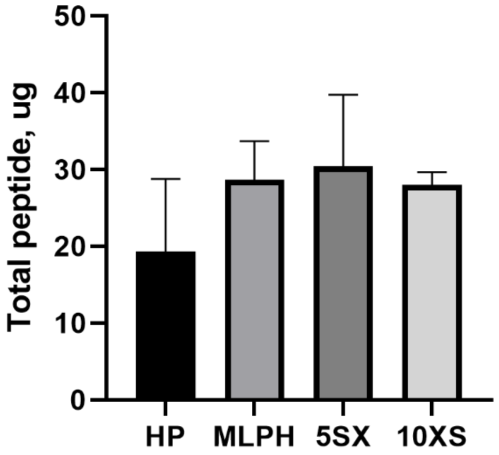

Figure S3. Total peptides in samples of liposome–plasma protein complexes assayed upon trypsinolysis by BCA (Pierce, USA) according to the manufacturer's recommendation. Mean values ± SD obtained for three replicate samples are reported.

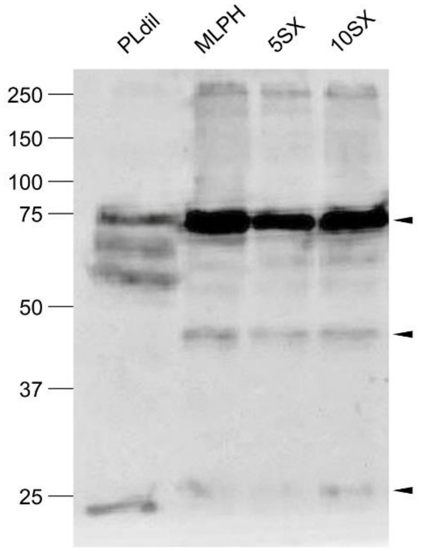

Figure S4. Western blot of liposomal protein corona components with anti-IgM antibodies. +, positive control, human plasma diluted 1250-fold; IgM is presented by heavy chain and its fragment due to SDS-PAGE reducing conditions.
